# Effects of Coatings on Antioxidant Enzyme Activities, Histopathology, and Transcriptome Profiles of Kidney Tissue in *Larimichthys crocea*

**DOI:** 10.3390/genes16040392

**Published:** 2025-03-29

**Authors:** Xuan Xu, Huayu Song, Lu Zhang, Chonghui Chen, Xiaoxu Zhang, Yiying Liu, Chao Li, Qiang Fu

**Affiliations:** 1 School of Marine Science and Engineering, Qingdao Agricultural University, Qingdao 266109, China; xuxuan989826@126.com (X.X.); c13325030859@163.com (C.C.); 17664020172@163.com (X.Z.); 15263348506@163.com (Y.L.); chaoli@qau.edu.cn (C.L.); 2 Qingdao Conson Oceantec Valley Development Co., Ltd., Qingdao 266237, China; song.huayu@163.com (H.S.); zhanglu1@qdgxjt.com (L.Z.)

**Keywords:** fish, aquaculture vessel coatings, RNA-seq, antioxidant enzyme activities, histology

## Abstract

**Background:** As an innovative approach to deep-sea aquaculture, fish farm vessels offer a dual benefit by alleviating the pressure on offshore fishing resources while providing an additional high-quality protein source. However, the potential impacts of vessel coatings on farmed fish remain poorly understood. **Methods:** In this study, to investigate the effects of vessel coatings on the large yellow croaker (*Larimichthys crocea*), we established four experimental groups with coating concentrations at 1-fold, 10-fold, 20-fold, and 80-fold levels. Antioxidant enzyme activities in kidney tissues were measured across all groups, while histological and transcriptome analyses were specifically conducted for the 1-fold and 80-fold concentration groups. **Results:** Firstly, significant alterations in antioxidant enzyme activity were observed in the 80-fold concentration group. Moreover, histological analysis demonstrated more severe pathological changes in kidney tissue at the higher concentration, including interstitial hemorrhage and tubular epithelial cell fatty degeneration. In addition, we identified 11,902 differentially expressed genes (DEGs) by high-throughput sequencing. KEGG pathway enrichment analysis revealed that the DEGs were predominantly involved in critical biological processes, including endoplasmic reticulum protein processing, oxidative phosphorylation, cytokine–cytokine receptor interactions, cell cycle regulation, DNA replication, and PPAR signaling pathways. Finally, the validation of nine selected DEGs through quantitative real-time PCR (qRT-PCR) showed significant correlation with RNA-Seq data, confirming the reliability of our transcriptome analysis. **Conclusions:** This study provides preliminary insights into the antioxidant stress response mechanisms of *L. crocea* to coating exposure and establishes a theoretical foundation for optimizing healthy fish farming practices in aquaculture vessels.

## 1. Introduction

In the past decades, the aquaculture industry has experienced remarkable growth, yet traditional practices predominantly confined to inland and offshore waters have encountered severe challenges, including excessive aquaculture densities, severe water pollution, and compromised aquatic product quality, all of which pose substantial risks to consumer dietary health [1,2,3]. In contrast, deep-sea aquaculture presents a promising alternative, offering the promotion of fish welfare and ecosystem benefits through superior water exchange mechanisms and the effective dispersion of waste across broader areas [4]. Mobile aquaculture vessels offer good self-propulsion and closed aquaculture environments; therefore, more and more aquaculture vessels are being used in deep-sea aquaculture [5]. Currently, to protect the inner walls of aquaculture vessel holds from seawater corrosion, solvent-free epoxy coatings are commonly applied. However, coatings have a limited lifespan in terms of corrosion resistance. Extended exposure to seawater may result in the release of harmful chemicals or residual solvents, which could have detrimental effects on farmed fish. Despite the growing body of research on the effects of environmental stressors on fish, there is a significant gap in understanding how coatings used on aquaculture vessels affected *L. crocea*. Previous studies primarily focused on the toxicity of specific chemicals or contaminants but largely ignored the effects of coatings, which could accumulate over time.

When exposed to environmental stimuli, farmed fish may generate excessive reactive oxygen species (ROS), leading to oxidative stress. This stress can damage DNA, proteins, and lipids, resulting in oxidative damage that may be harmful to the fish [6]. Changes in antioxidant enzyme activity can serve as an indicator of the physiological state of fish under varying environmental conditions, providing a useful parameter for assessing the extent of environmental stress in fish [7]. Superoxide dismutase (SOD), catalase (CAT), and peroxidase (POD) are key antioxidant enzymes that play a vital role in the antioxidant defense mechanism [8]. SOD converts O_2_^−^ into hydrogen peroxide (H_2_O_2_) and oxygen (O_2_), protecting the organism from free radical damage [9]. CAT and POD, on the other hand, catalyze the decomposition of H_2_O_2_ into molecular oxygen and water, thus effectively scavenging the accumulated H_2_O_2_ in the body and preventing the cells from suffering from its toxicity [10]. Additionally, malondialdehyde (MDA) is one of the end products of lipid peroxidation in the body, and its level can indirectly reflect the severity of free radical-induced damage [11]. Therefore, changes in antioxidant enzymes can be used to make a preliminary determination of the health effects of coatings on farmed fish.

The kidney is a critical organ in fish, playing an essential role in various physiological and regulatory processes, including fluid balance, osmoregulation [12], excretion of nitrogenous wastes, and maintaining acid–base equilibrium. Since most hazardous substances flow into the kidneys [13], kidney damage can serve as a reliable indicator of environmental pollution [14]. Oxidative stress and the generation of ROS are central to renal epithelial injury [15,16]. Furthermore, excessive ROS production may lead to DNA damage and apoptosis [17]. Kidney cell damage, such as Bowman’s edema, congestion, and tubular necrosis, can be observed in *L. crocea* exposed to p-nitrophenol (PNP) [18]. Similarly, in *Prochilodus lineatus*, the accumulation of TiO_2_ nanoparticles (TiO_2_-NPs) induces ROS generation, leading to tissue damage, including cellular and nuclear hypertrophy of kidney tubules, tubular degeneration, necrosis, and impaired kidney function [19]. It has been found that difenoconazole treatment damaged kidney tissue morphology and structure, enhanced creatinine (Cr) and blood urea nitrogen (BUN) levels, and led to inflammatory cells infiltration, threatening the kidneys of carp [20]. In addition to these physiological responses, environmental stresses induced by coatings immersion can significantly impact the transcriptome signature. The kidney, as a key organ in the fish immune system, plays an indispensable role in immune cell development, pathogen recognition, and immune response [21,22]. Transcriptome analysis of this organ can offer significant insights into the fish immune system, including the identification of differentially expressed genes and pathways that are involved in immune responses. For example, the transcriptome patterns of Nile tilapia (*Oreochromis niloticus*) under low-temperature stress reveal that low-temperature stress leads to kidney dysfunction and the down-regulation of immune-related pathways in the kidney, helping us to understand the molecular mechanisms of Nile tilapia’s response to environmental stresses [23]. However, information regarding the role of coatings in modulating immune activity at the transcriptional level in the kidneys of *L. crocea* is limited.

Large yellow croaker, known for its tender meat and rich nutritional value, is an important source of high-quality protein for coastal populations and a key economic fish along the southeast coast of China [24,25]. Deep-sea aquaculture vessels provide advantages such as protection from natural disasters like typhoons and red tides, which helps mitigate the economic losses caused by harsh environmental conditions in aquaculture [26]. Moreover, the use of intelligent management and feeding systems on aquaculture vessels can reduce fish mortality, improve fish quality, and increase yield. However, the impact of the coatings used in aquaculture vessels on *L. crocea* remains unclear. This study seeks to explore the impact of environmentally friendly aquaculture tank coatings on the kidneys of *L. crocea* by performing a comparative analysis at the antioxidant, histological, and transcriptome levels. The results of this work will increase our knowledge of the molecular mechanisms associated with the response of *L. crocea* to coatings.

## 2. Materials and Methods

### 2.1. Ethics Statement

All experiments were performed in accordance with local government regulations, and all procedures involving fish were conducted following the ethical guidelines established by the Institutional Animal Care and Use Committee of Qingdao Agricultural University, Qingdao, China.

### 2.2. Experimental Animals, Coating Immersion, and Tissue Collection

The coatings used in this experiment were environmentally friendly aquaculture tank coatings composed of epoxy resin, curing agent, pigmented filler, and other components, specifically designed for the aquaculture tanks of the “Guoxin No. 1” fish farming vessel. The water volume of the aquaculture tank in “Guoxin No. 1” is 4992 m^3^, with an inner wall coating area of 1530 m^2^. The total amount of coatings used in the tank is approximately 189.7 L, corresponding to 0.038 L of coatings per cubic meter of aquaculture water. To simulate the actual coating concentration in the aquaculture tank (referred to as the 1-fold concentration treatment group), coating samples (each containing 10.98 mL of coatings) were immersed in 0.288 m^3^ of aquaculture water, resulting in a 1-fold concentration of 0.038 L/m^3^. Ultimately, through extensive pre-experimentation, the 1-fold concentration coating group (38.125 mL/m^3^), the 10-fold coating concentration group (381.25 mL/m^3^), the 20-fold coating concentration group (762.5 mL/m^3^), and the 80-fold treatment group (3050 mL/m^3^) were selected for experimentation.

A total of 162 *L. crocea* (mean weight: 100.6 ± 10.4 g; mean body length: 17.5 ± 0.3 cm) were obtained from Zhejiang Guoxin (Taizhou) Fishery Company Limited. The environmentally friendly coatings were provided by Qingdao Marine Chemical Research Institute. The fish were temporarily reared in the aquaculture system at the Marine Science and Engineering, Qingdao Agricultural University, for one week. During this period, the main water parameters were carefully controlled: water temperature was maintained between 18 °C and 25 °C, pH ranged from 7.5 to 8.0, and the dissolved oxygen concentration was kept above 5 mg/L. The experiment was divided into 2 concentration groups, including the 1-fold concentration group and 80-fold concentration group, each with three replicates. Following the temporary rearing period, the fish were randomly assigned to 9 tanks, with 18 fish per tank. The culture experiment was conducted for one week and adequate oxygen supply was ensured.

Random samples were collected at 0 h, 12 h, 24 h, 48 h, 72 h, and 96 h following coatings immersion. At each time point, nine fish per group were randomly selected (three from each tank) and quickly anesthetized using 100 mg/L MS-222 (Solarbio, Beijing, China) to isolate kidney tissues. These tissues were immediately snap-frozen in liquid nitrogen and stored at −80 °C for subsequent transcriptome sequencing, antioxidant enzyme activity analysis, and RT-qPCR. For histological analysis, kidney tissues from the 1-fold and 80-fold concentration groups were fixed at 24 h and 48 h in 4% paraformaldehyde (PFA) (Solarbio, Beijing, China) for further staining.

### 2.3. Biochemical Analysis

The activities of CAT and SOD were measured using assay kits obtained from the Nanjing Jiancheng Bioengineering Institute, while POD activity and MDA concentration were determined using kits from Beijing Soleilbao Science and Technology Company Limited. For the assays, approximately 0.1 g of kidney tissue was collected, and cold saline was added at a 1:9 mass/volume ratio. The tissue was then homogenized on ice, followed by centrifugation at 3000× *g* for 15 min at 4 °C to obtain the supernatant for analysis.

### 2.4. Histological Analysis

Kidney tissues from 3 biological replicates per group were fixed in 4% paraformaldehyde for 24 h, followed by dehydration through a graded ethanol series, xylene transparency and paraffin embedding. Finally, each sample was sliced discontinuously 3 times using a RM26 Leica microtome (Leica, Wetzlar, Germany) to obtain 5–6 μm sections. After staining with hematoxylin and eosin (H & E), the sections were examined under a microscope (Zeiss, Oberkochen, Germany) [27].

### 2.5. RNA Extraction, Library Construction, and Sequencing

The extraction of total RNA was carried out using Trizol^®^ Reagent sourced from Invitrogen (Carlsbad, CA, USA), adhering strictly to the manufacturer’s instructions. Subsequently, the integrity of the RNA was examined on 1% agarose gels. The Nanodrop 2000 instrument from Thermo Fisher Scientific (Boston, FL, USA) was employed to ascertain both the quality and quantity of RNA. For the purpose of cDNA library construction, only those samples exhibiting an A260/280 ratio and an A260/230 ratio exceeding 1.8, along with an RNA integrity number (RIN) above 7.0, were deemed eligible for selection. The confirmation of RNA concentrations was additionally conducted using a NanoPhotometer spectrophotometer from IMPLEN (Westlake Village, CA, USA). Three bioreplicates at each time point were analyzed, and 15 libraries were obtained for sequencing.

Oligo(dT) magnetic beads sourced from Qiagen (Hilden, Germany) were utilized to isolate Poly(A)-mRNA from the pooled total RNA. Subsequently, the mRNA was fragmented into smaller segments through the application of fragmentation buffer. Utilizing random hexamer primers and M-MuLV reverse transcriptase, these short fragments were employed as templates for the synthesis of the first-strand complementary DNA (cDNA). Synthesis of the second-strand cDNA ensued, which was subsequently subjected to end repair, 3′ adenylation, and adapter ligation processes. The targeted cDNA fragments underwent purification via agarose gel electrophoresis and were subsequently enriched through PCR amplification. Ultimately, sequencing of the cDNA libraries was conducted on the BGISEQ DNBSEQ-T7 platform provided by BGI Inc. (Shenzhen, China), yielding 150 bp paired-end reads as a result.

### 2.6. Read Mapping and Differential Expression Analysis

The quality of the sequencing data was evaluated using FastQC (http://www.bioinformatics.babraham.ac.uk/projects/fastqc/, accessed on 20 May 2024) to ensure that high-quality clean reads would be obtained. Trimmomatic (version 0.32) [28] was employed to process the raw reads by eliminating adapter sequences and ambiguous nucleotides. Post-filtering, only those read pairs with both sequences exceeding 30 base pairs in length were retained for further analysis. The filtered reads were then aligned to the *L. crocea* reference genome [29] using Hisat2 (version 2.0.5), allowing up to 5% mismatches in the mapped length and requiring at least 90% base alignment to the genome. Read counts were extracted from the mapping profiles using HTSeq-count in the recommended mode [30].

The level of gene expression was assessed by calculating Fragments Per Kilobase of transcript per Million mapped reads (FPKM). To identify differentially expressed genes (DEGs), the DESeq R package (1.18.0) was utilized, applying criteria of a fold change ≥2 and an adjusted *p*-value from the false discovery rate (FDR) < 0.05 [31]. Venn diagrams and volcano plots were used to visually represent the DEGs at different time points.

### 2.7. GO and KEGG Analyses

Enrichment analyses based on Gene Ontology (GO) and Kyoto Encyclopedia of Genes and Genomes (KEGG) were performed utilizing the GOseq R package [32] and the KOBAS platform (accessible at http://www.genome.jp/kegg/, accessed on 15 June 2024), respectively. Statistical significance was evaluated using the hypergeometric test, and a *p*-value < 0.05 was considered the threshold for identifying significant enrichment GO terms and KEGG pathways between groups.

### 2.8. Experimental Validation by Quantitative Real-Time PCR

Kidney tissue was used to extract total RNA, employing the Trizol reagent (Invitrogen, Carlsbad, CA, USA) according to the manufacturer’s guidelines. Subsequently, the extracted RNA was converted into cDNA using the PrimeScript™ RT reagent Kit (Takara, Otsu, Japan). Real-time quantitative PCR (RT-qPCR) was conducted on the CFX96 real-time PCR detection system (Bio-Rad Laboratories, Hercules, CA, USA) using SYBR^®^ Green Realtime PCR Master Mix Premix Ex Taq (Takara, Japan), adhering strictly to the manufacturer’s protocol. The primers utilized in the experiment were designed through PrimerQuest (https://sg.idtdna.com/PrimerQuest/Home, accessed on 8 October 2024), as detailed in Table S1. The relative expression level was calculated using the 2^−ΔΔCt^ (*n* = 3) method [33], with β-actin as an internal normalization control [34]. Three replicates were performed for each sample.

### 2.9. Statistical Analysis

The analysis of significant differences among samples was conducted using one-way ANOVA, followed by post hoc tests employing the least significant difference (LSD) method for further exploration. Data are presented as mean ± SE. All statistical analyses were performed using IBM SPSS 20.0 (SPSS, Chicago, IL, USA). *p* < 0.05 was considered statistically significant, while *p* < 0.01 was considered highly significant. GraphPad Prism version 8.0 software was used for plotting.

## 3. Results

### 3.1. Enzyme Activity Levels

**Figure 1:**
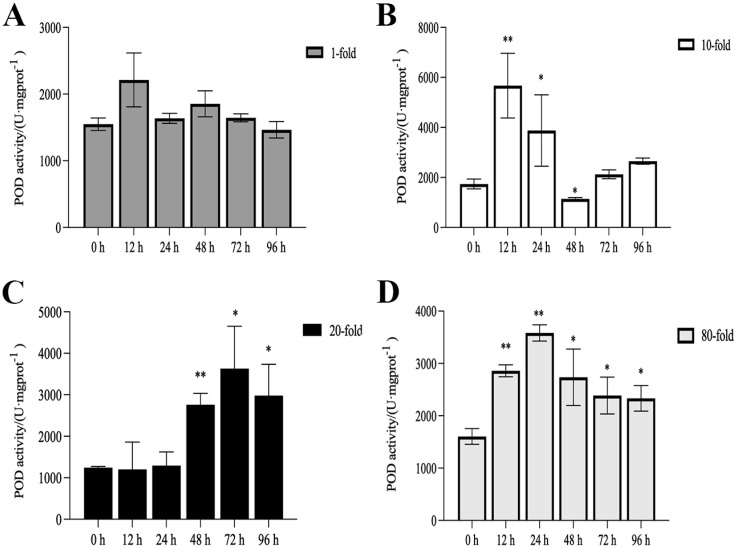
Activity of peroxidase (POD) in the kidneys of *L. crocea* under (**A**) 1-fold, (**B**) 10-fold, (**C**) 20-fold, and (**D**) 80-fold coating concentration. * means *p* < 0.05; ** means *p* < 0.01.

**Figure 2:**
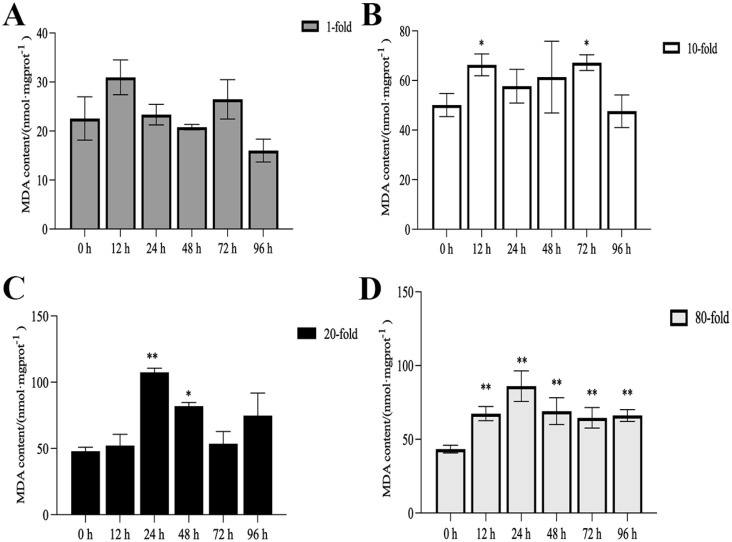
Contents of malondialdehyde (MDA) in the kidneys of *L. crocea* under (**A**) 1-fold, (**B**) 10-fold, (**C**) 20-fold, and (**D**) 80-fold coating concentration. * means *p* < 0.05; ** means *p* < 0.01.

**Figure 3:**
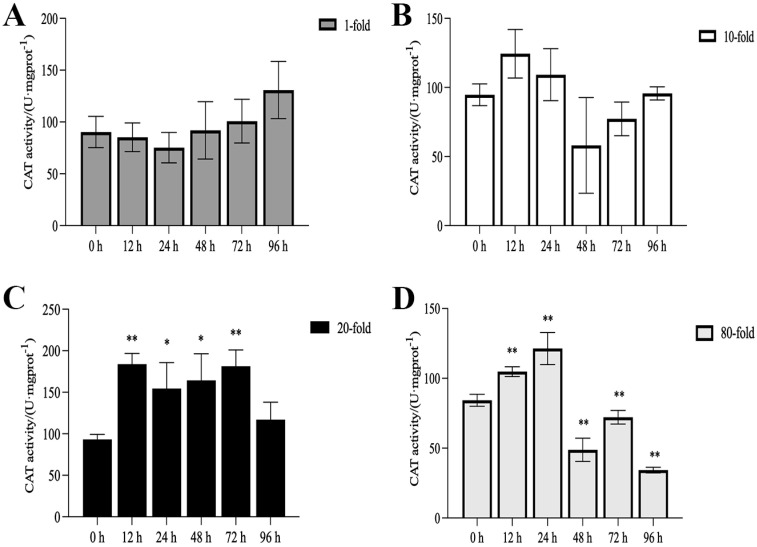
Activity of catalase (CAT) in the kidneys of *L. crocea* under (**A**) 1-fold, (**B**) 10-fold, (**C**) 20-fold, and (**D**) 80-fold coating concentration. * means *p* < 0.05; ** means *p* < 0.01.

**Figure 4:**
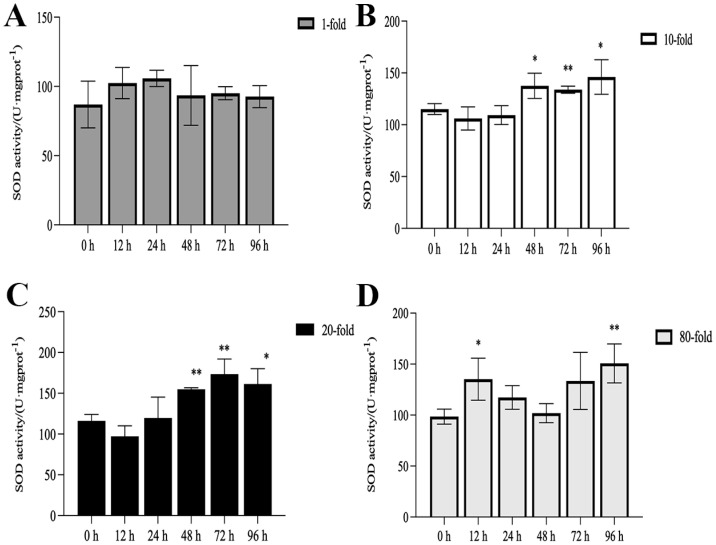
Activity of superoxide dismutase (SOD) in the kidneys of *L. crocea* under (**A**) 1-fold, (**B**) 10-fold, (**C**) 20-fold, and (**D**) 80-fold coating concentration. * means *p* < 0.05; ** means *p* < 0.01.

The activities of SOD, CAT, and POD, as well as MDA content, are illustrated in Figure 1, Figure 2, Figure 3 and Figure 4. Compared with the control group, there was no significant difference in the activities of CAT, POD, and SOD in the 1-fold concentration group (*p* > 0.05). The activities of POD in the 1-fold and 80-fold concentration groups showed a tendency to increase and then decrease, and the activity of POD in the 80-fold concentration group was significantly higher than that in the control group (*p* < 0.05) (Figure 1A,D). A similar situation was observed in the MDA content of the 80-fold concentration group (Figure 2D). At 96 h, CAT activity in the 80-fold concentration group was significantly lower than that of the control group (*p* < 0.05) (Figure 3D). In contrast, SOD activity in the same concentration group was significantly higher than that of the control group (*p* < 0.05) at 96 h (Figure 4A–D). At 96 h, there was no significant difference in CAT and POD activities in the 10-fold concentration group as compared to the control group (*p* > 0.05) (Figure 1B and Figure 3B). The 20-fold concentration group showed significantly higher POD, CAT and SOD activities compared to the control group at 72 h (*p* < 0.05) (Figure 1C, Figure 3C and Figure 4C).

### 3.2. Histological Observation

**Figure 5:**
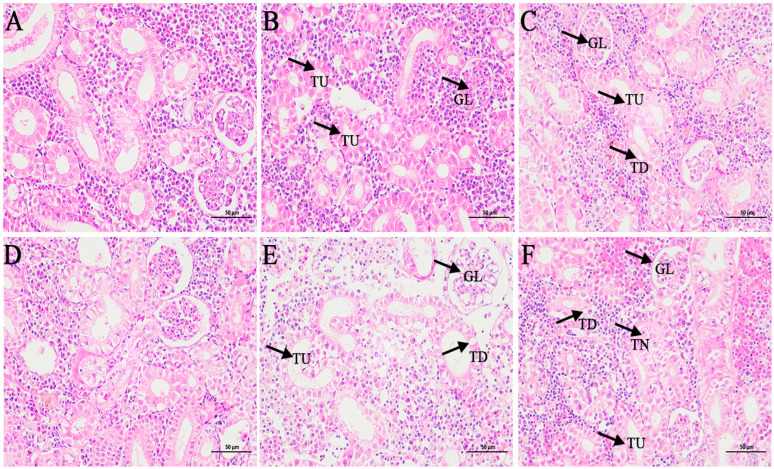
Effects of coatings on kidney histopathological features of *L. crocea.* (**A**,**D**) Kidney tissue structures in the control group; (**B**,**E**) kidney tissue structure at 24 h and 48 h in the 1-fold concentration group; (**C**,**F**) kidney tissue structure at 24 h and 48 h in the 80-fold concentration group. GL: glomerulus; TU: renal tubule; TN: renal tubular epithelial cell necrosis; TD: fatty degeneration of renal tubular epithelial cells.

Kidney tissues of the control group (Figure 5A,D) showed normal physiological morphology with well-defined tubular and glomerular structures. Fatty degeneration of renal tubular epithelial cells was observed in kidney tissue after 24 and 48 h of exposure to 1-fold concentration (Figure 5B,E). At 80-fold concentration (Figure 5C,F), interstitial hemorrhage, fatty degeneration of tubular epithelial cells, and tubular epithelial cell necrosis were observed.

### 3.3. Sequencing and Mapping of Expressed Short Reads

Expressed short reads were obtained from the kidneys of *L. crocea* through RNA-seq analysis, comparing the control group at 0 h with coating-treated groups (1_24 h, 1_48 h, 80_24 h, and 80_48 h). A summary of the results obtained after filtering out low-quality reads, rRNA reads, reads containing adapters, and reads with more than 10 unknown nucleotides is presented in Table S2, showing a total of 802,154,400 clean reads. The sequence data were deposited in the NCBI Sequence Read Archive (SRA) with Bioproject number PRJNA1,212,533. Following filtering, the sequencing data exhibited high quality, as evidenced by Q20 values ranging from 98.56% to 99.23% and Q30 values between 95.71% and 97.34%. Additionally, an average of 92.09% of the sequences were successfully mapped to the *L. crocea* reference genome, yielding a total of 738,732,195 mapped reads (Table S2).

### 3.4. Analysis of Differentially Expressed Genes

**Figure 6:**
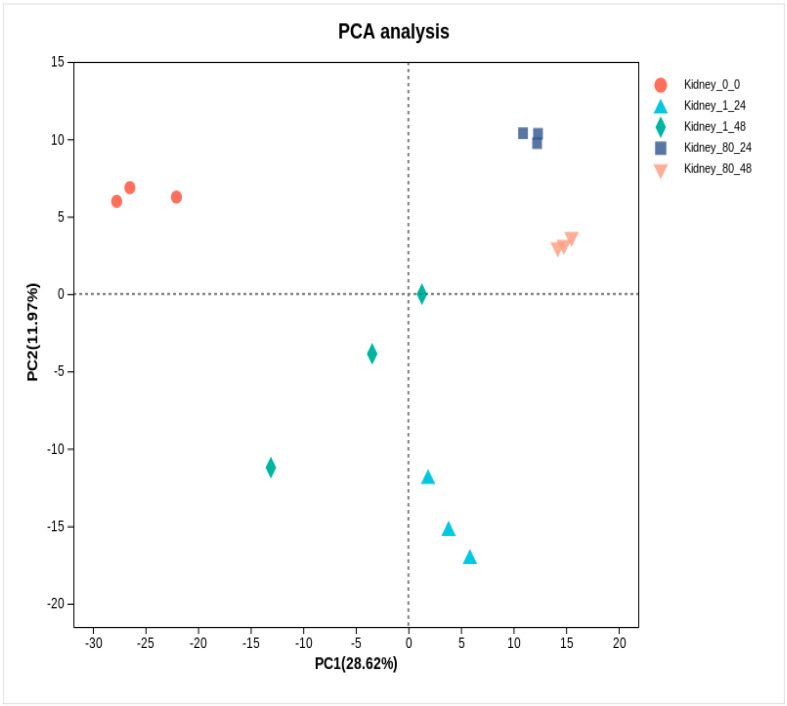
Principal component analysis (PCA) of kidney transcriptome at 24 h and 48 h after coating immersion.

**Figure 7:**
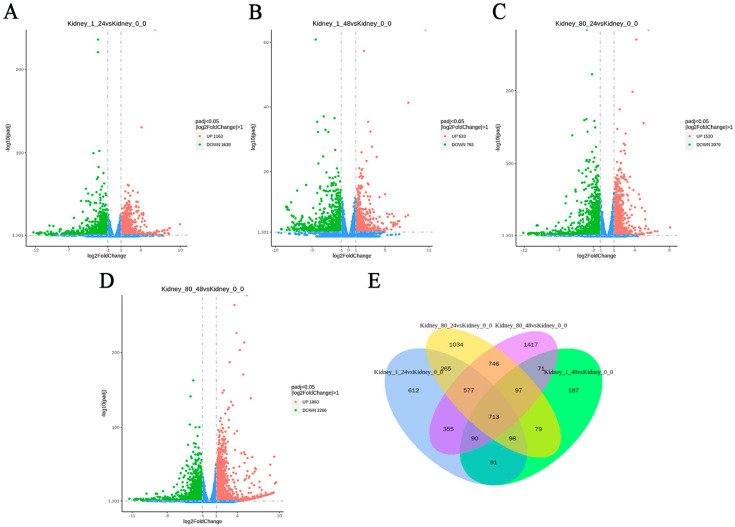
Analysis of DEGs in *L. crocea* kidney at different time points after coating immersion displayed through volcano plot and Venn diagram. (**A**,**B**) Volcano plots of DEGs in *L. crocea* kidney at 24 h and 48 h in the 1-fold concentration group, respectively. (**C**,**D**) Volcano plots of DEGs in *L. crocea* kidney at 24 h and 48 h in the 80-fold concentration group, respectively. The x-axis represents the value of Log_2_ (fold change), and the y-axis represents the value of –Log_10_ (FDR). Red and green spots represent significantly up-regulated and down-regulated genes, respectively (|log_2_ (fold change)| > 1), while blue spots represent no difference in gene expression. (**E**) Venn diagram.

Principal component analysis (PCA) revealed a distinct separation between the control group and the coating-treated group (Figure 6). To identify DEGs in the kidneys of *L. crocea*, pairwise comparisons were made between the control group and coating-treated groups (1-fold and 80-fold) at two time points (24 h and 48 h). As shown in Table 1 and Figure 7A–D, a total of 2801 DEGs (1163 up-regulated, 1638 down-regulated) were identified in the 1-fold concentration group at 24 h, and 1426 DEGs (633 up-regulated, 793 down-regulated) were identified at 48 h. For the 80-fold concentration group, 3609 DEGs (1530 up-regulated, 2079 down-regulated) were identified at 24 h, and 4066 DEGs (1860 up-regulated, 2206 down-regulated) were identified at 48 h. Furthermore, the Venn diagram (Figure 7E) illustrates a substantial overlap of DEGs between the experimental and control groups, with 713 DEGs common across all four comparisons.

### 3.5. Analysis of Expression Signatures, Clustering, and GO Analysis

**Figure 8:**
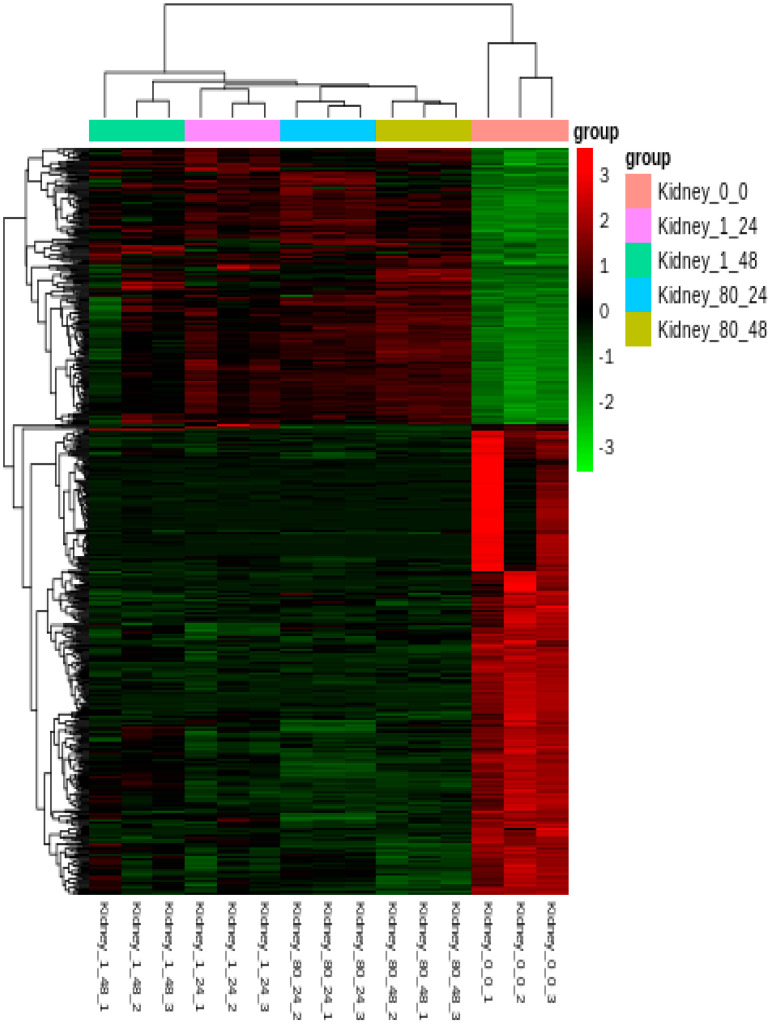
Heatmap display of hierarchical clustering of all DEGs in the kidneys of *L. crocea* at different time points after coatings immersion. DEGs are displayed in rows, while samples are displayed in columns.

**Figure 9:**
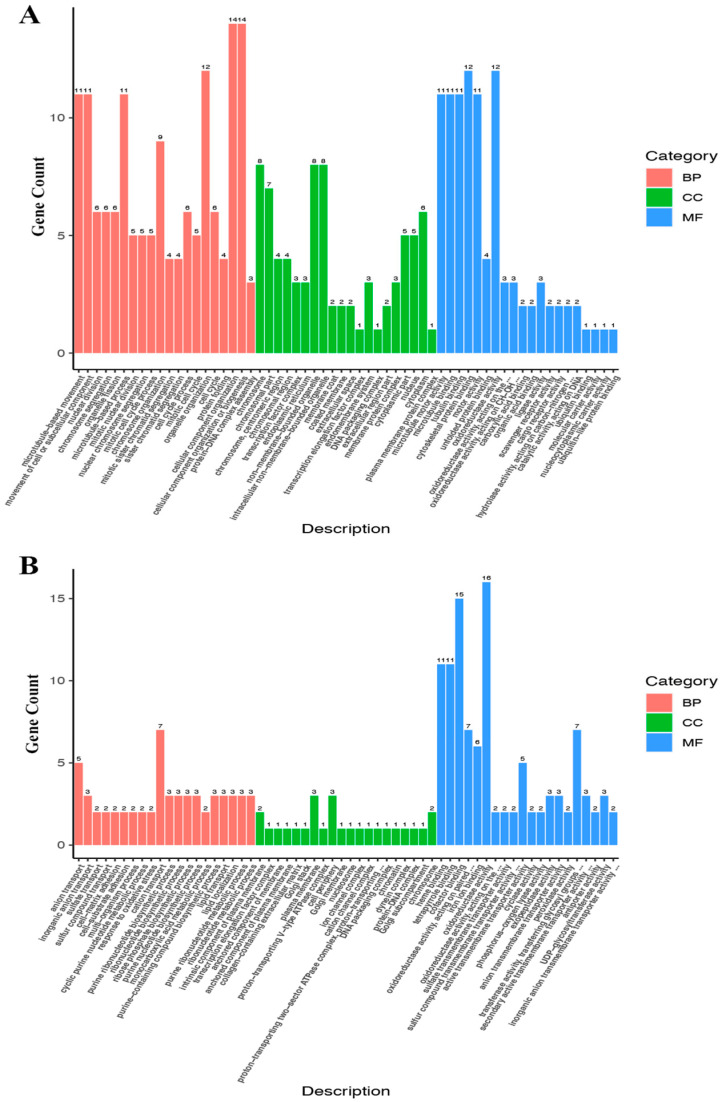
Classification map of secondary DEG entries. The x-axis represents the second-level GO entry, and the y-axis represents the number of DEGs in the GO entry. (**A**) GO analysis of up-regulated DEGs; (**B**) GO analysis of down-regulated DEGs.

Hierarchical cluster analysis demonstrated significant differences in the expression patterns of DEGs between untreated and treated groups (Figure 8). GO annotation was used to classify the transcripts into functional groups based on GO categories. For the up-regulated DEGs (Figure 9A), in the molecular function (MF) category, the most represented genes were related to cytoskeletal protein binding (GO:0,008,092) and oxidoreductase activity (GO:0,016,491). In the biological process (BP) category, the most prominent terms included cellular component organization (GO:0,016,043) and cellular component organization or biogenesis (GO:0,071,840). In the cellular component (CC) category, the most frequent genes were related to the chromosome (GO:0,005,694), non-membrane-bounded organelles (GO:0,043,228), and intracellular non-membrane-bounded organelles (GO:0,043,232). For the down-regulated DEGs (Figure 9B), the most enriched GO terms in the BP category were biological regulation (GO:0,065,007) and regulation of biological processes (GO:0,050,789). In the MF category, the most prevalent terms were receptor activity (GO:0,004,872), molecular transducer activity (GO:0,006,009), and signal transducer activity (GO:0,004,871). In the CC category, the most enriched GO terms included extracellular region (GO:0,005,576) and extracellular matrix (GO:0,030,198).

### 3.6. Signaling Pathway Analysis

**Figure 10:**
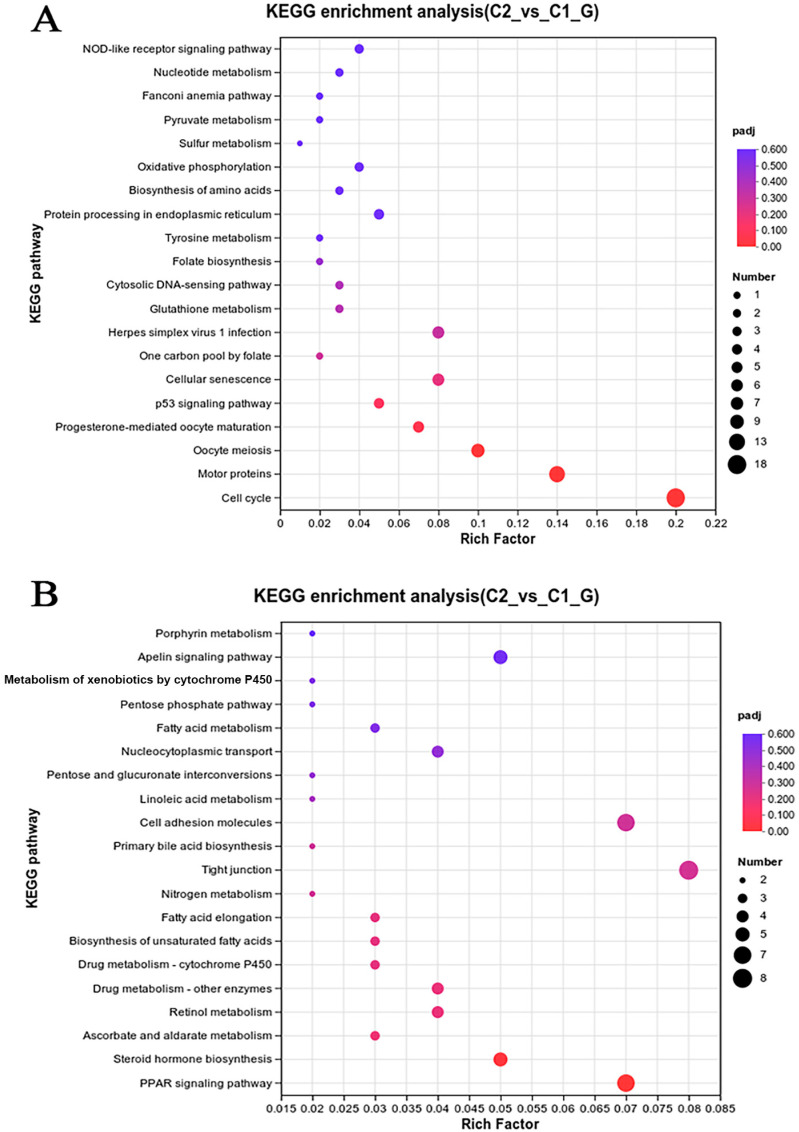
Pathway analysis of DEGs in the kidneys of *L. crocea* between control group and coating-treated groups based on the KEGG database. The vertical coordinates indicate the KEGG channel. The horizontal coordinates indicate the Rich factor. The larger the Rich factor, the greater the degree of enrichment. The larger the point, the greater the number of differential genes enriched in the pathway. (**A**) Pathway analysis of up-regulated DEGs; (**B**) pathway analysis of down-regulated DEGs.

**Figure 11:**
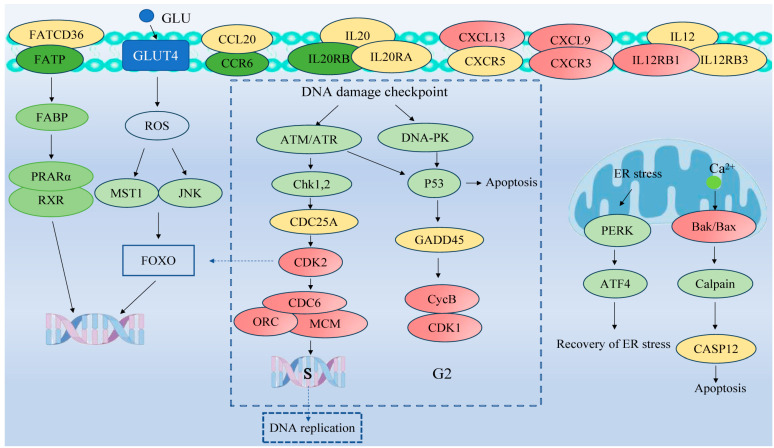
Representative pathway analysis of DEGs in *L. crocea* kidney between control group and coating-treated groups based on KEGG database. Red and green represent up-regulated and down-regulated genes, respectively, while yellow represent no difference in gene expression.

To investigate the pathways affected by coating immersion in *L. crocea*, DEGs were compared against the KEGG database for pathway enrichment analysis. The top 40 KEGG biological pathway classifications are presented in Figure 10. For the up-regulated DEGs (Figure 10A), significant enrichment was observed in several key pathways, including the cell cycle (lco04,110), motor proteins (lco04,814), DNA replication (lco03,030), protein processing in the endoplasmic reticulum (lco04,141), oocyte meiosis (lco04,114), the p53 signaling pathway (lco04,115), and oxidative phosphorylation (lco00,510). For the down-regulated DEGs (Figure 10B), enrichment was observed in the PPAR signaling pathway (lco03,320) and cytokine–cytokine receptor interaction (lco04,060), among other pathways, indicating changes in lipid metabolism and immune signaling. The DEGs were classified into six functional pathways, including oxidative phosphorylation, cell cycle, DNA replication, protein processing in the endoplasmic reticulum, the PPAR signaling pathway, and cytokine–cytokine receptor interaction, by enrichment analysis, pathway analysis, manual annotation, and literature review (Table S3). These pathways are critical for metabolic and immune responses. The schematic diagram (Figure 11) illustrates the hypothesized pathways involved in metabolic and immune responses in *L. crocea* kidney after coatings treatment. 

### 3.7. Validation of RNA-Seq Results by qRT-PCR

**Figure 12:**
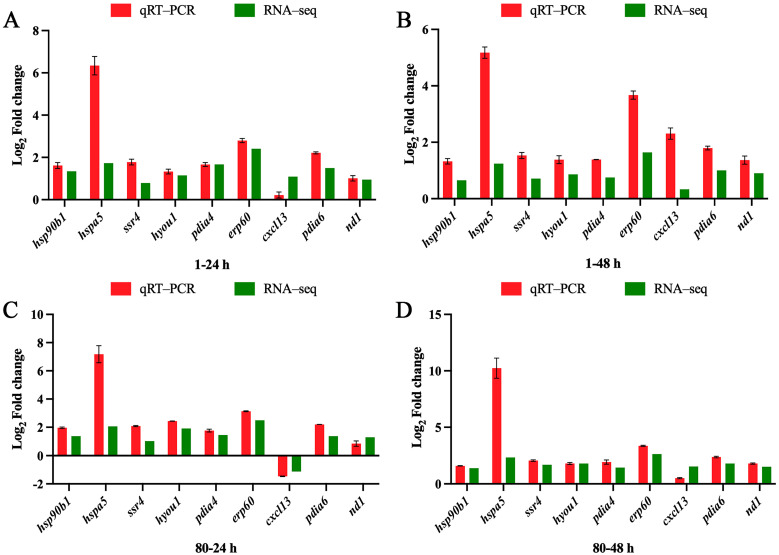
Validation of RNA-Seq results using qPCR. Comparison of DEG expression data between RNA-seq (green bars) and qRT-PCR (red bars). The x-axis presents the time points, and the y-axis presents the Log_2_ Fold change in DEG expression. (**A**,**B**) Changes in gene expression in the 1-fold concentration group at 24 h and 48 h compared with the control group; (**C**,**D**) Changes in gene expression in the 80-fold concentration group at 24 h and 48 h compared with the control group.

RT-qPCR was employed to validate the reliability of the RNA-seq results. Nine genes—*hsp90b1*, *hspa5*, *ssr4*, *pdia4*, *pdia6*, *hyou1*, *erp60*, *dnajb11*, and *nd1*—were selected from the RNA-seq data for the confirmation of their mRNA expression levels. The RT-qPCR results showed a strong correlation with the RNA-seq data, confirming the consistency and accuracy of the sequencing data (Figure 12).

## 4. Discussion

The present study investigated the differences in the antioxidant enzyme activities, histology, and transcriptome of the kidney tissues of *L. crocea* under different concentrations of coating treatments. The objective was to establish a theoretical foundation for the rational application of coatings in aquaculture vessels and to promote healthy marine fish aquaculture.

### 4.1. Antioxidant Enzyme Activity

When fish are exposed to environmental stress, they experience a stress response, which often results in increased production of ROS within the body [35]. Antioxidant enzymes play indispensable roles in regulating ROS generation and lipid peroxidation [36]. In this study, the changes in antioxidant enzymes in the 1-fold concentration treatment group (Figure 1,2 and 4)were not significantly different from those in the control group (*p* > 0.05), indicating that the 1-fold concentration of coatings did not cause any damage to the organism of the *L. crocea*. However, the antioxidant enzyme activities in the 80-fold concentration treatment group changed to different degrees as the coating immersion concentration increased. The SOD enzyme activity in the 80-fold concentration group was significantly higher at 12 h (Figure 4D), indicating that the stress caused by the coating activated the antioxidant enzyme system at an early stage. This result is similar to the increase in SOD activity seen in juvenile turbot *Scophthalmus maximus* under high-temperature stress [37]. In the present study, POD and CAT activities showed a trend of increasing and then decreasing in the 80-fold concentration group (Figure 1D and Figure 3D), suggesting that when the antioxidant system is unable to eliminate or neutralize excess ROS, the risk of oxidative damage increases due to the accumulation of lipid peroxides, which in turn may reduce the enzyme activities. MDA is a product of lipid peroxidation in the organism, and its content can indirectly reflect the severity of the organism’s cells under the attack of free radicals [38,39]. In this study, the MDA content (Figure 2D) in the 80-fold concentration group exhibited an initial increase followed by a gradual decrease over time. Notably, MDA levels remained significantly higher than those in the control group at 96 h. Increased MDA content indicates enhanced oxidative stress in organisms, suggesting that the environmental stress induced by the coating promoted lipid peroxidation in the large yellow croaker. The subsequent reduction in MDA levels implies that the organisms partially alleviated lipid peroxidation through peroxidase-mediated enzymatic regulation.

### 4.2. Histological Pathology

In fish, similar to higher vertebrates, the kidney plays a crucial role in maintaining a stable internal environment, and due to the high blood flow through the kidney, lesions detected in this organ can serve as signs of environmental pollution [40]. In the present study, by observing the histopathological sections of the kidneys of the large yellow croaker, it could be found that the kidneys of the 1-fold coating concentration-treated group did not show significant damage, whereas necrosis of tubular epithelial cells and interstitial hemorrhage were observed in the 80-fold concentration-treated group. Changes in the organization of renal tubular epithelial cells and glomeruli have been observed in fish after exposure to toxic substances such as pesticides [41]. A similar phenomenon was reported by Ortiz et al., who observed necrosis, detachment, and vacuolization of renal tubular epithelial cells in the kidneys of lindane-exposed fish [14]. An acute exposure to Cd at different concentrations in European sea bass (*Dicentrarchus labrax*) showed that tubular epithelial cell damage was directly related to the dose and exposure time of the toxicant [42].

### 4.3. Transcriptome Analysis

In this study, a total of 802,154,400 clean reads were obtained, and 738,732,195 reads were successfully aligned to the reference genome of the large yellow croaker. Additionally, 11,902 DEGs were identified in the kidneys of large yellow croaker after coating immersion. Among them, 1163 and 633 genes were significantly up-regulated and 1638 and 793 genes were significantly down-regulated after 24 h and 48 h of coating immersion, respectively, at 1-fold concentration. Meanwhile, 1530 and 1860 genes were significantly up-regulated and 2079 and 2206 genes were significantly down-regulated after 24 h and 48 h of coating immersion, respectively, at 80-fold concentration. Enriched GO terms were classified as biological processes (BPs), cellular components (CCs), and molecular functions (MFs). Among biological processes, DEGs were mainly enriched in GO terms such as anion transport, cellular component organization or biogenesis, and cation transport. In molecular functions, DEGs were mainly enriched in GO terms such as cytoskeletal protein binding, cofactor binding, and oxidoreductase activity. In cell components, DEGs were mainly enriched in GO terms such as chromosome, plasma membrane, and cell periphery. To verify the results of RNA-Seq, we performed RT-qPCR for genes with different expression patterns. The results of RT-qPCR were significantly correlated with those of RNA-Seq, indicating that RNA-Seq was reliable for gene expression analysis (Figure 12).

In the present study, based on a combination of GO enrichment analysis and literature reviews, DEGs can be divided into several representative pathways, such as cytokine–cytokine receptor interaction, protein processing in the endoplasmic reticulum, oxidative phosphorylation, cell cycle, DNA replication, and the PPAR signaling pathway. Those putative pathways are illustrated in Figure 10 and Figure 11 and Table S3. Below is a discussion on the putative functional roles and interactions of these signaling pathways.

#### 4.3.1. Oxidative Phosphorylation

Oxidative phosphorylation is the primary mechanism for the production of adenosine triphosphate (ATP) in organisms, serving as an essential source of energy that sustains cellular functions and accounts for approximately 90% of the cellular energy requirements [43]. This pathway also plays a crucial role in regulating and maintaining metabolic homeostasis [44,45]. In fish, substantial evidence suggests that the activity of oxidative phosphorylation adapts in response to various environmental factors, such as metabolic capacity, dietary composition, temperature fluctuations, and exposure to environmental contaminants [46]. Additionally, in response to environmental stress, fish organisms generate significant amounts of ROS. ROS are byproducts of mitochondrial oxidative phosphorylation and biological metabolism, and they are key indicators of the organism’s response to stress. During oxidative phosphorylation, a small proportion of consumed oxygen is converted into ROS. However, when ROS levels exceed certain thresholds, they can impair the efficiency of oxidative phosphorylation, leading to oxidative damage to critical biomolecules [47]. Consequently, energy metabolism and ROS production are two interconnected facets of the organism’s stress response, with their balance playing a pivotal role in maintaining cellular integrity under stressful conditions [48]. In our study, exposure to coatings led to oxidative damage in the kidney tissue of *L. crocea*, triggering a significant up-regulation of key oxidative phosphorylation-related genes, including NADH dehydrogenase subunits (*nd1*, *nd2* and *nd6*) and ATP synthase subunit (*atp6*). This up-regulation suggests an acceleration of NADH oxidation and ATP synthesis, likely as a compensatory mechanism to repair cellular damage caused by oxidative stress. The increased ATP production may reflect the high energy demand necessary to counteract the detrimental effects of oxidative stress and maintain cellular function. Our findings align with previous research showing that oxidative phosphorylation is highly responsive to environmental stressors. For example, ammonia exposure can cause liver tissue damage and an up-regulation of genes related to oxidative phosphorylation in largemouth bass (*Micropterus salmoides*), which increases energy expenditure through oxidative phosphorylation and alters the nutrient metabolic pathways of *M. salmoides* in response to counter-environmental effects [49]. Similarly, copper exposure in large yellow croaker leads to mitochondrial ultrastructural damage, heightened ROS production, and a decrease in oxidative phosphorylation efficiency [50]. Exposure to ammonia induced oxidative stress and resultant protein damage in juvenile *Eriocheir sinensis*, significantly enhanced the oxidative phosphorylation process, and produced more ATP to cope with high-concentration-ammonia stress [51]. Under low-temperature stress, seawater (SW)-acclimated milkfish responds to adverse environmental effects by regulating oxidative phosphorylation pathways and metabolic gene expression to generate more energy [52]. Under acute high-temperature stress, juvenile *Acrossocheilus wenchowensis* adapts to acute high-temperature stress through oxidative phosphorylation-mediated energy metabolism by increasing ATP production to meet higher energy demands and maintaining intracellular redox balance to alleviate oxidative stress [53]. Overall, these findings underscore the crucial role of oxidative phosphorylation in mediating the stress response to environmental challenges, maintaining cellular energy balance, and mitigating oxidative damage in *L. crocea* under coating exposure.

#### 4.3.2. Cytokine–Cytokine Receptor Interaction

Cytokines are considered to be important extracellular signaling molecules that mediate intercellular communication during immune responses [54]. Cytokines can generally be categorized into six primary groups: interferons, interleukins (ILs), the transforming growth factor-β (TGF-β) superfamily, colony-stimulating factors (CSFs), the tumor necrosis factor (TNF) superfamily (also known as TNFSF), and chemokines. Chemokines, a specific subclass of cytokines, play a pivotal role in regulating leukocyte chemotaxis [55]. Among the various pathways involving cytokines, the cytokine–cytokine receptor interaction pathway stands out as particularly significant. Cytokines serve as crucial regulators of cell growth, immune modulation, and angiogenesis [56]. Notably, cytokines can induce inflammatory responses by binding to their specific receptors on target cells [57]. In our study, exposure to coatings led to significant changes in the expression of key genes involved in the cytokine–cytokine receptor interaction pathway, particularly those associated with chemokines and interleukins. The expression levels of *ccl28*, *il-20rb*, and *il-7r* were down-regulated at both 24 h and 48 h post-exposure, with the most pronounced effects observed under the 80-fold concentration treatment. Among these, *ccl28* exhibited the most significant down-regulation, with log_2_FC values ranging from −0.58 to −1.47, suggesting its potential role in the stress response to coating exposure (Table S3). The chemokine *ccl28*, known for its antimicrobial and immunomodulatory properties, also promotes mucosal homing of T and B lymphocytes [58]. High expression of *ccl28* has been associated with enhanced mucosal defense, whereas its suppression can lead to increased vulnerability to infections. Similarly, the decreased expression of *il-20rb* and *il-7r* suggests potential disruptions in cytokine signaling. *il-20rb* is part of the *il-20* receptor complex involved in immune regulation and epithelial tissue homeostasis, while *il-7r* is crucial for T-cell development and survival [59,60]. The down-regulation of these genes could indicate an overall immunosuppressive effect, potentially reducing the ability of *L. crocea* to mount an effective immune response against environmental stressors.

#### 4.3.3. Protein Processing in the Endoplasmic Reticulum

The endoplasmic reticulum (ER) is responsible for protein folding and trafficking and exhibits a high degree of sensitivity to alterations in intracellular homeostasis, as well as extracellular stimulatory signals [61]. Endoplasmic reticulum stress (ERS), including the accumulation of unfolded proteins and protein misfolding, usually occurs due to the disturbances of intracellular environment, which ultimately leads to damage to endoplasmic reticulum function [62,63]. In this study, many DEGs were enriched in protein processing in the endoplasmic reticulum. At 1-fold concentration, the log_2_FC values of all genes detected at 48 h were lower than the values detected at 24 h, while at 80-fold concentration, the majority of genes, including *hspa5, ssr4*, and *pdia6,* exhibited higher log_2_FC at the 48 h time point. The increased expression of *hspa5* suggested that the *L. crocea* kidney cells responded to the imbalance of protein homeostasis caused by coating exposure by enhancing protein folding ability. Furthermore, *hspa5* could mitigate the damage to the ER caused by oxidative stress through inhibiting ROS generation or enhancing antioxidant enzyme activity [64]. In addition, studies of the response of *M. salmoides* to HS in the protein processing in the endoplasmic reticulum pathway showed that a large number of genes related to heat shock response, especially *hsp90a1, hsp90aa1, dnajb1,* and *dnajb6*, were significantly up-regulated [65,66]. In rainbow trout (*Oncorhynchus mykiss*), the expression levels of *hsp90aa*, *hsp90ab*, and *hsp40* at 24 °C were higher than those at 18 °C after heat stress [67]. Among them, the activation of *hsp90s* could serve to halt protein aggregation, thereby facilitating the acceleration of endoplasmic reticulum homeostasis [68].

#### 4.3.4. PPAR Signaling Pathway

The peroxisome proliferator-activated receptor (PPAR) belongs to the superfamily of nuclear receptor transcription factors, which encompasses three subtypes: PPARα, PPARβ/δ, and PPARγ [69]. These receptors regulate a variety of physiological processes such as lipid and lipoprotein metabolism, glucose homeostasis, cell proliferation and differentiation, cell cycle progression, inflammation, and extracellular matrix remodeling [70,71,72]. PPARs are also implicated in many kidney pathophysiological conditions, including diabetic nephropathy and glomerulosclerosis [73]. In the kidney, the expression of PPARα is predominantly observed in the proximal tubules and medullary thick ascending limbs, whereas lower levels of expression are detected in glomerular mesangial cells. Notably, PPARα has been shown to reduce the inflammatory response in LPS-treated mesangial cells, suggesting its potential role in glomerular diseases [74,75]. Furthermore, PPARα is essential during microbial infections, as its activation may modulate metabolism, thereby altering immune function and cellular activity [76]. In immune cells, PPARα is specifically present in peripheral mononuclear immune cells, such as macrophages, and is expressed in large amounts in them [77]. PPARγ plays an important role in various biological processes, including adipogenesis, inflammatory reactions, cell growth regulation, cell differentiation, oxidative stress, and endoplasmic reticulum (ER) stress [78,79]. In the kidney, PPARγ with expressed at lower levels in the glomeruli and kidney microvasculature, whereas it is primarily expressed in medullary collecting ducts [80]. PPARγ is known to promote sodium and fluid reabsorption by enhancing the expression of epithelial sodium channels (ENaC) in kidney collecting ducts [81]. In our study, exposure to coatings resulted in a significant down-regulation of key genes involved in the peroxisome proliferator-activated receptor (PPAR) signaling pathway, particularly cpt1b and fabp1. The expression of fabp1 exhibited the most substantial decline, with log_2_FC values ranging from −4.30 to −9.19 (Table S3). This suggests a severe disruption in fatty acid metabolism, as *fabp1* plays a crucial role in fatty acid transport and intracellular lipid homeostasis. Concurrently, the down-regulation of *cpt1b* indicates impaired fatty acid β-oxidation, which may lead to lipid accumulation and disturbances in energy metabolism [82,83]. Given the critical role of the PPAR pathway in renal function, these molecular changes suggest that coating exposure may contribute to renal tubular injury in *L. crocea*. Our findings align with previous studies demonstrating that environmental stressors can suppress PPAR signaling, leading to metabolic dysfunction. For instance, heat stress in pigs resulted in the down-regulation of PPAR pathway genes, including cpt1b and fabp3, impairing fatty acid β-oxidation and energy metabolism [84]. In conclusion, these findings indicate that coatings exposure disrupts lipid metabolism and energy homeostasis in *L. crocea* by suppressing the PPAR signaling pathway, which may ultimately contribute to renal dysfunction and metabolic stress in affected fish.

#### 4.3.5. Cell Cycle and DNA Replication

The cell cycle is divided into four distinct phases: G1 (pre-DNA synthesis), S (DNA replication), G2 (post-DNA replication), and M (cell division) [85]. G1 phase, which is characterized by direct cell division, is also known as the post-mitotic pre-synthesis phase. The S phase represents the phase of DNA synthesis in the cell cycle. The G2 phase (pre-mitotic or post-synthesis phase) can be considered true division, when the cell is ready to divide into two cells. Lastly, during the M- or mitosis-phase division, the doubled DNA in the chromosomes is separated [86]. Loading of the six MCM subunits (Mcm2-7) usually occurs at the end of mitosis to form the pre-replication complex [87]. Cyclin-dependent kinase 1 (*cdk1*), originally identified as a key regulator of the cell cycle, has emerged as a multifunctional protein kinase with a broad range of roles. In addition to its regulatory functions in the cell cycle, *cdk1* is also involved in DNA damage repair and various other cellular processes essential for cell survival. In our study, exposure to coatings resulted in a significant up-regulation of key genes involved in cell cycle regulation and DNA replication, particularly *cdk1*, *mcm2*, *mcm6*, and *mcm7* (Table S3). The increased expression of these genes suggests that coating exposure may accelerate cell cycle progression in *L. crocea* kidney cells, potentially as a compensatory response to cellular stress. Among them, *cdk1*, a pivotal regulator of cell cycle transitions, exhibited elevated expression, indicating its role in facilitating cell cycle progression under coating-induced stress. In *Saccharomyces cerevisiae*, *cdk1* is necessary and sufficient for cell cycle regulation by phosphorylating a large number of substrates to coordinate cell cycle events [88]. Jones et al. demonstrated that *cdk1* promotes adhesion complex formation and increases the cell adhesion area from G1 to the S phase [89]. Furthermore, *cdk1* has been shown to influence tumor progression by inducing a dysregulation of the cell cycle [90]. Notably, *mcm2*, *mcm6*, and *mcm7*, which encode components of the minichromosome maintenance (MCM) complex responsible for DNA replication initiation, were also up-regulated, suggesting enhanced DNA replication activity [91].

DNA replication is important for passing genetic information to daughter cells and offspring, and all organisms have mechanisms to protect the authenticity of DNA replication [92]. One key player in these processes is replication protein A (RPA), a single-strand DNA-binding protein consisting of three subunits (RPA1, RPA2, and RPA3). RPA is involved in DNA repair, meiosis, and replication, and it also activates cellular responses to DNA damage [93]. Our study revealed that exposure to coatings led to a significant up-regulation of *rpa1* and *rpa3* gene expression, with *rpa1* showing a notable increase (log_2_FC ranging from 1.24 to 2.04) (Table S3). This suggests that the coating treatment induced a cellular response associated with DNA replication and repair, potentially as a protective mechanism against DNA damage. Similar findings have been reported in other organisms; for example, in *Oryza sativa*, γ irradiation led to increased expression of *rpa3a* and *rpa3b*, demonstrating the role of RPA in DNA replication and repair under environmental stress conditions [92]. The increased expression of RPA-related genes suggests that kidney cells in *L. crocea* were actively responding to coating-induced DNA stress.

## 5. Conclusions

The potential effects of coatings on *L. crocea* were investigated through antioxidant enzyme activity, histology, and transcriptome analyses of kidney tissue. The results indicate that 80-fold concentration of coatings significantly enhances kidney antioxidant enzyme activity in the short term. Additionally, necrosis of renal tubular epithelial cells was observed in the kidney tissue structure. High-throughput sequencing identified 11,902 differentially expressed genes (DEGs). KEGG pathway enrichment analysis indicated that many DEGs were enriched in pathways such as cell cycle, the PPAR signaling pathway, and DNA replication. This study provides preliminary theoretical insights into the mechanisms of antioxidant stress response in *L. crocea* induced by coatings and offers guidance for the healthy farming of fish in aquaculture vessels.

## Figures and Tables

**Table 1:** Summary of DEGs in *L. crocea* kidney at different time points after immersion coatings with a cutoff of FDR corrected *p*-value < 0.05 and |log_2_ (fold change)| > 1.

DEGs	Kidney_1_24	Kidney_1_48	Kidney_80_24	Kidney_80_48	Kidney_1_24
Up-regulated	1163	633	1530	1860	1163
Down-regulated	1638	793	2079	2206	1638
Total	2801	1426	3609	4066	2801

## Data Availability

The raw data supporting the conclusions of this article will be made available by the authors on request. The raw RNA-seq data (Accession no. PRJNA1212533) were uploaded to NCBI.

## Supplementary Materials

The following supporting information can be downloaded at https://www.mdpi.com/article/10.3390/genes16040392/s1, Table S1. Primers used for RT-qPCR. Table S2. Summary of RNA-seq of *L. crocea* kidney transcriptomes post immersion coatings. Table S3. Representative DEGs and their log2FC valuein the kidney of *L. crocea* at different time points after coatings immersion involved in key molecular pathways.
