# Supplementary material for: Effects of Coatings on Antioxidant Enzyme Activities, Histopathology, and Transcriptome Profiles of Kidney Tissue in Larimichthys crocea
Source: Genes (Basel). 2025 Mar 29;16(4):392. doi: 10.3390/genes16040392 (PMC12026950; doi:10.3390/genes16040392)
Supplement: Supplementary file 1 [file genes-16-00392-s001.zip › genes-3531706-supplementary.pdf]

Supplementary Table S1. Primers used for RT-qPCR.

Gene	Gene name	Primer sequences
β-actin	Beta-actin	F: TGGCATCACACCTTCTACAAC
		R: ACGACCAGAGGCATACAGG
hsp90b1	heat shock protein 90 beta family member 1	F: TCACCACATGGCTGAAGGAC
		R: AGCCATACTGACTGGCAACC
hspa5	heat shock protein family A member 5	F: CGAGGAGAAGATTGAGTGGATG
		R: CGTAGAGCTTGCTGATGATAGG
ssr4	signal sequence receptor subunit 4	F: GGCCAGGATGTTGGCAAGTA
		R: GTTTCTCTGGGCCTTACGCA
pdia4	protein disulfide isomerase family A member 4	F: GAGGTGCTGTCAGACTTTGT
		R: CGACCTTAACTGGTCCTTTGT
pdia6	protein disulfide isomerase family A member 6	F: GTGTGGATGGTGGAGTTCTT
		R: CGAACTTTGCCCTTGGTTTG
hyou1	hypoxia up-regulated 1	F: ACCGGGAGGTCAACTACTTA
		R: TGGCCTTGCTGTTCTTCTC
erp60	Endoplasmic reticulum resident protein 60	F: TGGATCTGTGGCAGGTTAAAT
		R: CTGCTCCTCCTTCATCTTCTTC
dnajb11	DnaJ heat shock protein family member B11	F: GAGAGGACCTTGGAGGTAGAA
		R: ACTTTGATGCGGAAACGTAGA
nd1	NADH dehydrogenase subunit 1	F: CTTCACCCTTCAAACCTTCAAC
		R: CTCTGCCAGTGTGGAGATATAC

Supplementary Table S2. Summary of RNA-seq of *L. crocea* kidney transcriptomes post immersion coatings.

Sample Name	Clean Reads	Clean Bases	Clean GC (%)	Clean Q20 (%)	Clean Q30 (%)	Total mapped reads (%)
Kidney_0_0_1	49,746,138	7.46 G	48.66	98.83	96.40	45,565,619 (91.6%)
Kidney_0_0_2	39,415,544	5.91 G	48.69	98.56	95.71	36,187,490 (91.81%)
Kidney_0_0_3	41,124,530	6.17 G	48.73	98.69	96.07	37,769,085 (91.84%)
Kidney_1_24_1	55,577,536	8.34 G	49.49	98.97	96.89	51,515,891 (92.69%)
Kidney_1_24_2	79,422,964	11.91 G	48.87	98.60	95.81	73,338,180 (92.34%)
Kidney_1_24_3	77,232,724	11.58 G	48.99	99.23	97.34	71,696,558 (92.83%)
Kidney_1_48_1	54,178,470	8.13 G	47.80	98.92	96.75	49,263,602 (90.93%)
Kidney_1_48_2	48,951,500	7.34 G	49.22	98.67	96.02	45,291,577 (92.52%)
Kidney_1_48_3	42,100,854	6.32 G	48.5	98.74	96.24	38,794,515 (92.15%)
Kidney_80_24_1	58,871,590	8.83 G	49.41	99.05	97.14	54,280,427 (92.2%)
Kidney_80_24_2	43,806,100	6.57 G	49.21	98.77	96.32	40,658,372 (92.81%)
Kidney_80_24_3	76,713,202	11.51 G	49.65	99.03	97.07	70,844,301 (92.35%)
Kidney_80_48_1	40,468,204	6.07 G	49.05	98.73	96.21	37,103,016 (91.68%)
Kidney_80_48_2	51,812,020	7.77 G	49.09	98.92	96.78	47,215,509 (91.13%)
Kidney_80_48_3	42,733,024	6.41 G	49.06	98.85	96.54	39,208,053 (91.75%)
Total	802,154,400	120.32 G				738,732,195 (92.09%)

Supplementary Table S3. Representative DEGs and their log2FC valuein the kidney of L. crocea at different time points after coatings immersion involved in key molecular pathways.

**Gene name**	**log2FC**			
	**1-24 h**	**1-48 h**	**80-24 h**	**80-48 h**
**Cytokine-cytokine receptor interaction**				
cxcl13	1.09	0.33	-1.13	1.54
ccl28	-1.97	-2.99	-5.50	-5.19
il-20rb	-1.85	-1.77	-1.82	-1.16
il-12b	-0.26	-0.49	-1.57	-2.08
il-7r	-0.35	-0.60	-0.60	-1.02
cxcl13	1.09	0.33	-1.13	1.54
**Protein processing in endoplasmic reticulum**				
hsp90b1	1.34	0.65	1.38	1.40
hspa5	1.73	1.24	2.06	2.34
ssr4	0.79	0.71	1.03	1.69
pdia4	1.67	0.75	1.46	1.44
ddost	1.14	0.88	1.25	1.70
pdia6	1.50	1.00	1.38	1.81
erp60	2.41	1.64	2.49	2.65
dnajb11	1.23	0.91	1.96	1.92
hyou1	1.15	0.86	1.91	1.80
**Oxidative phosphorylation**				
nd1	0.95	0.90	1.30	1.52
atp6	1.14	1.04	1.60	1.64
nd6	0.68	0.93	1.45	1.16
nd2	0.84	0.62	1.35	1.30
**Cell cycle**				
cdk1	1.90	1.35	1.82	2.10
mcm6	1.12	0.74	1.43	1.57
mcm7	0.89	0.60	1.13	1.29
mcm2	1.10	0.80	1.24	1.53
**PPAR signaling pathway**				
cpt1b	-1.18	-0.53	-0.36	-1.12
fabp1	-4.50	-5.21	-9.19	-4.30
**DNA replication**				
rpa1	1.81	1.24	1.97	2.04
pcna	1.20	0.75	1.22	1.44
rpa3	1.53	0.84	1.35	1.64
